# Yield Response and Calibration of Critical Potassium Levels in Soil, Leaves, and Fruit Pulp of “Royal Gala” and “Fuji Suprema” Apples

**DOI:** 10.3390/plants15121866

**Published:** 2026-06-16

**Authors:** Leandro Hahn, Clori Basso, Jean M. Moura-Bueno, Luiz Carlos Argenta, Gilberto Nava, Moreno Toselli, Corina Carranca, Danilo Eduardo Rozane, Gustavo Brunetto

**Affiliations:** 1 Agricultural Research and Rural Extension Agency of Santa Catarina (Epagri), Caçador 89501-032, Brazil; leandrohahn@epagri.sc.gov.br (L.H.);; 2 Soil Science Department, Federal University of Santa Maria (UFSM), Santa Maria 97105-900, Brazil; brunetto.gustavo@gmail.com; 3 Embrapa Clima Temperado, Caixa Postal 403, Pelotas 96010-971, Brazil; 4 Department of Horticulture and Forestry, University of Bologna, 40126 Bologna, Italy; 5 Instituto Nacional de Investigação Agrária e Veterinária, 2780-157 Lisboa, Portugal; 6 Faculty of Agricultural Sciences of Vale do Ribeira, São Paulo State University (Unesp), Registro 11900-000, Brazil; danilo.rozane@unesp.br

**Keywords:** *Malus domestica* Borkh., nutritional status, K in apples, nutrient reference concentration, quantile regression models

## Abstract

The yield of apple trees as a function of potassium fertilization and the critical levels (CLs) and sufficiency ranges (SRs) of K in the soil, leaves, and fruits were determined in two experiments (two orchards) in four crop seasons. Plants of “Royal Gala” and “Fuji Suprema” cultivars were treated with 0, 50, 100, 150, or 200 kg K_2_O ha^−1^ year^−1^. Potassium was applied annually during the bud swelling phase and onto the soil surface in the projection of the plant canopy, without incorporation. Critical levels and SR were estimated by Bayesian segmented quantile regression models. The cultivar factor was the main source of variation in fruit yield, K concentration in leaves and pulp, and K exported by apples. The crop season was the second factor with the greatest contribution to apple yield and K concentrations in leaves. When data from all crop seasons and orchards were pooled, yield did not vary by K treatments. The concentration of K in the leaf and fruit pulp also did not change as a function of the K dose with grouped data. For fruit production, the CL of K in the soil was 170 mg dm^−3^ for both cultivars; 17.8 g kg^−1^ and 15.8 g kg^−1^ in leaf for “Fuji Suprema” and “Royal Gala”, respectively; 1150 mg kg^−1^ and 1080 mg kg^−1^ in fruit pulp for “Fuji Suprema” and “Royal Gala”, respectively. The lack of response to K fertilization indicates that the trees were operating within a nutritional plateau. Consequently, we recommend that K fertilization in subtropical apple orchards be guided strictly by soil and plant analysis. For orchards exceeding the soil critical level of 170 mg dm^−3^ and leaf concentrations of 17.8 g kg^−1^ and 15.8 g kg^−1^ in leaf for “Fuji Suprema” and “Royal Gala”, respectively, and 1150 mg kg^−1^ and 1080 mg kg^−1^ in fruit pulp for “Fuji Suprema” and “Royal Gala”, respectively, K applications may be reduced or temporarily withheld under similar high-K soil conditions, provided that soil and plant nutritional status are regularly monitored. This management strategy ensures high yields and more efficient and sustainable nutrient management.

## 1. Introduction

Tropical and subtropical soils normally do not have a sufficient amount of potassium (K) available to meet the nutrient demand by apple trees (*Malus domestica* Borkh.) [1]. Therefore, it is necessary to conduct applications of potassium fertilizers, where the need can be established considering the K concentration available in soils and the cation exchange capacity of soils (CTC_pH7.0_) [1]. Soils with higher CTC_pH7.0_ values can adsorb greater amounts of K [2,3]. Potassium concentration in leaves and yield expectations can be complementary variables when deciding on the need for K application and doses. However, whenever possible, the most appropriate K doses to be applied in apple orchards should be established in calibration experiments [4]. These experiments must take into account official recommendations for apple production. But they must also meet as much as possible the soil and climate conditions and management adopted in commercial orchards, such as cultivars and rootstocks, planting density, plant training system, sources, forms, time of fertilizer application, etc.

Potassium is one of the nutrients required in the greatest quantity for the growth and development of apple trees and fruits [1,5], in addition to reducing the incidence of fungal diseases [6]. Furthermore, adequate K nutrition contributes to the quality of apple fruits, as K is a cofactor of enzymes associated with the synthesis of anthocyanins [7], a pigment that gives the red color to fruits [8]. On the other hand, excess K in the soil can reduce Ca and Mg absorption [9,10,11] and reduce their concentrations in the plant [12,13]. This can alter the K/Ca, K/Mg, and K+Mg/Ca relationships in fruits [11], increasing the incidence of physiological disorders, such as bitter pit [14,15,16]. However, adequate doses and levels of available K in soils can contribute to obtaining adequate levels of K in apple trees, which can be diagnosed by analyzing the leaves [17,18]. Adequate K content in leaves positively reflects the increase in fruit yield [1] and the activity of the roots and enzymes involved in the C and N metabolism [7].

Soils in southern Brazil, where apples are grown, have high levels of organic matter and clay and, therefore, high cation exchange capacity (CEC) [2]. Due to this feature, the official K recommendations for apple orchards in the production phase establish K applications in a single plot during the bud swelling phase, and K is applied to the soil surface in the projection of the plant canopy, without incorporation. 

Responses to K fertilization may be different depending on cultivars. This is because cultivars may have different morphological characteristics of the root system, which also determines the variables related to nutrient absorption efficiency, such as the Michaelis–Menten constant (Km), maximum speed (Vmax), and minimum concentration (Cmin) [19]. Furthermore, cultivars may have different vegetative vigor and yield, also affected by rootstock type, which affects the demand for K and even the amount of exported K [20,21,22]. In addition, cultivars in conditions of low K availability in the soil solution may have strategies/mechanisms, such as exudation of organic compounds and modification of pH values in the rhizospheric soil [23,24]. This can increase the solubility of minerals that contain K, including in non-exchangeable forms [25], increasing the availability of exchangeable K [26,27].

Climatic variables, such as temperature, winter cold hours, humidity, solar radiation, and precipitation, can also affect the yield and nutritional status of apple trees [28,29]. Lower rainfall volumes can reduce soil moisture and, consequently, the approach of ions to the external surface of the roots, such as K [30]. In the soil, most of the K is supplied via diffusion, a mechanism very dependent on the water content in the soil. Therefore, water stress can reduce K concentrations in leaves and fruits and even the productivity [8,13].

Thus, establishing critical levels (CLs) and sufficiency ranges (SRs) of K in soils and leaves is an important strategy to maximize plant production and maintain fruit quality. This tool allows us to precisely define the real need and quantity of nutrients to be applied, especially K, which is absorbed, accumulated, and exported in greater quantities in fruit trees [11,20,22]. Critical level and SR values can be obtained using the boundary line method [31], which considers that the best performance of a population is described by a line on the edge of any body of data and occurs whenever there is a cause-and-effect relationship between two variables. This method uses the linear-plateau function [32]—also known as piecewise regression—with two segments separated by an inflection point. From this point onwards, no increase in yield is observed, even with the increase in nutrient concentration in the soil and leaves. Bayesian segmented quantile regression (BSQR) models associate nutrient concentrations and crop yield [32,33]. Studies that address the use of models to estimate CL and SR of nutrients in soils and leaves are still scarce [33,34,35], especially those carried out with fruit trees, such as apple trees.

Therefore, the establishment of nutrient CL and SR in soils and leaves can help in the use of more appropriate fertilizer doses [36], especially when CLs are generated for specific cultivars [35,36], which results in maximizing yield, but also maintaining fruit quality. The definition of CL and SR values contributes to reducing the potential for soil and water contamination, and other types of losses due to excessive fertilizer applications [34,35]. The apple trees “Gala”, “Fuji”, and their clones—the two most planted apple cultivars in southern Brazil—differ in several aspects of their growth and development [36]. Therefore, it is reasonable to assume that the CL of K in the soil and leaves for the production of these cultivars are different. This study aimed to (i) evaluate the yield of “Royal Gala” and “Fuji Suprema” apples grown in a subtropical climate subjected to K fertilization for four crop seasons and (ii) determine critical levels (CLs) and sufficiency ranges (SRs) of K in the soil, leaves, and fruits.

## 2. Materials and Methods

### 2.1. Characterization of the Experimental Area

**Figure 1:**
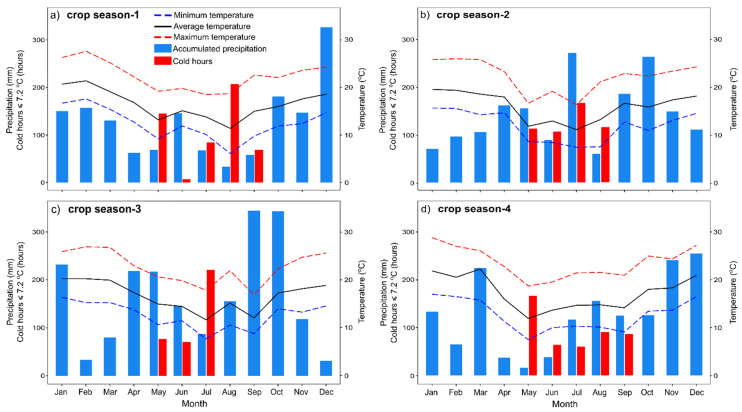
Minimum, mean, and maximum temperatures (°C), monthly accumulated precipitation (mm), and number of hours less than or equal to 7.2 °C during crop season—1 (**a**), 2 (**b**), 3 (**c**), and 4 (**d**).

This study was carried out in four crop seasons (from 2003 to 2006) in two orchards, called Orchards A and B, in the municipality of *Fraiburgo*, state of *Santa Catarina*, southern region of Brazil (27.107123 S and 50.914899 W for Orchard A and 26.972091 S and 50.902439 W for Orchard B). The climate in the region is humid subtropical (Cfa) [37], characterized by mild temperatures and precipitation with little variation throughout the year. Data on climate variables observed over the years are presented in Figure 1. The soil in both orchards was classified as Typic Hapludalf [38]. The orchards’ soil had spontaneous vegetation composed of *Trifolium repens*, *Lolium multiflorum*, and *Paspalum notatum*.

### 2.2. Experiment Setup

In 2002, two experiments were installed: experiment 1 (Orchard A) and experiment 2 (Orchard B). Each orchard was formed by cultivars “Royal Gala” and “Fuji Suprema”, both grafted onto the M9 rootstock. All trees were trained as tall spindles. Orchard A was implemented in 2000 with a planting density of 2857 plants ha^−1^ for both cultivars. In Orchard B, “Royal Gala” was planted in 1997, with a density of 3570 plants ha^−1^, and “Fuji Suprema” in 2000, with a density of 2500 plants ha^−1^. The two locations were chosen for representativeness of this study, seeking to have a greater number of individuals for evaluation. The two orchards are 30 km apart and have the same soil, altitude, and climate characteristics, as well as the same management of cultural practices. The only factor that changes between orchards is the spacing between plants.

The experiments with the application of K doses began in 2002, when the orchards were already in full production, that is, from the third year after implementation. The experiment used a randomized block design, with four replications, with each replication consisting of 10 apple trees, with the following K_2_O doses: 0, 50, 100, 150, and 200 kg K_2_O ha^−1^ year^−1^, referring to 0, 41.5, 83, 124.5, and 166 kg K ha^−1^ year^−1^. The dose of 100 kg K_2_O ha^−1^ year^−1^ is an average dose recommended in [2] to replenish the K exported by fruits in orchards in full production. The K source used was potassium chloride (60% K_2_O), applied annually during the bud swelling phase. K was applied to the soil surface in the projection of the plant canopy, without incorporation, for four consecutive crop seasons (from 2003 to 2006). Annually, 100 kg K ha^−1^ as ammonium nitrate (33% N) was also applied. Half of the N was applied at bud swelling, and the second half in post-harvest. Other nutrients throughout the experimental period were not applied. Pest and disease control management practices were carried out following regional recommendations for apple trees [39]. The orchards were not irrigated, relying solely on regional rainfall distribution.

In the second half of January, of the four evaluated crop seasons, 50 fully expanded, healthy leaves were collected per plot [2] in the four quadrants of the plants, in the middle third of the shoot growth in the current year. The leaves were dried in a forced air oven at 65 °C until constant mass, ground in a Wiley mill, and stored in a paper bag. Soil samples were collected in the 0–0.20 and 0.20–0.40 m layers before applying the treatments in 2002 (Table 1) and in the winters of 2004 and 2006.

In all treatments, manual fruit thinning was performed to maintain a crop load of one to two fruits per cluster. Harvesting was scheduled according to the starch–iodine index, maintained between 3 and 5 for “Royal Gala” and 4 and 6 for “Fuji Suprema” to ensure commercial maturity. Flowering occurred from late September to early October across all seasons. For “Royal Gala” and “Fuji Suprema”, harvests were performed in February and March, respectively, with yield recorded by fruit count and average weight determination. Also, twenty medium-sized fruits (130 g to 150 g) were sampled for mineral evaluation. Annual trunk diameter was measured at harvest in all experimental trees, at 0.3 m above the graft union.

### 2.3. Soil, Leaf, and Fruit Pulp Analysis

The leaf concentration of K was determined after nitro-perchloric digestion [40], and K readings were taken on an atomic absorption spectrophotometer (PerkinElmer, Waltham, MA, USA, Analyst 2000).

The mineral concentrations in the fruit were determined in the whole fruit (pulp + peel): wedge-shaped longitudinal slice (1 cm thick), without the central part of the carpel, extracted from each fruit. Subsequently, the concentration of K was determined based on the same methodology used to determine the leaf concentration. To obtain the K export by the apple tree, the following equation was used: K = concentration of K in the fruit (g kg^−1^) × average fruit weight (g). Soil samples were prepared, and K levels were extracted using Mehlich-1 [41]. Potassium was determined using a flame photometer (Digimed, BM-62, São Paulo, Brazil).

### 2.4. Climatic Characteristics

Meteorological data from the experiment evaluation period were obtained at the meteorological station of the *Empresa de Pesquisa Agropecuária e Extensão Rural de Santa Catarina* [42], located in the municipality of *Fraiburgo* (SC). The following variables were compiled for each evaluated station: Minimum, mean, and maximum temperatures, mean precipitation, relative air humidity, chill hours (below 7.2 °C), and chill units [43], as per the number of hours accumulated, according to the modified North Carolina model [43], in the winter period (June to September).

The accumulated precipitation and mean air temperature values ranged between the four crop seasons (Figure 1). In the first crop season, unfavorable weather conditions for apple cultivation are observed, such as rainfall and cold times below average, mainly in the months of April, May, June, August, and September (Figure 1a). This behavior is also observed in the fourth crop season (Figure 1d).

### 2.5. Statistical Analysis

Initially, the analysis of variance components was conducted to quantify the percentage contribution of each source of variation (doses of K, crop season, cultivar, orchard, and interactions) on the response of crop yield and its components and K concentration in leaves and pulp. The relative contribution of each source of variation was calculated by dividing the estimated variance component of each factor by the total variance (i.e., the sum of all variance components, including the residual variance), and expressing the result as a percentage. This statistical procedure was performed using the “VCA” package in the R statistical programming environment, which estimates variance components based on restricted maximum likelihood. This statistical procedure was carried out using the “VCA” package of the R statistical programming language [44]. The ANOVA was performed using the R package “ExpDes.pt”. Whenever the null hypothesis (equal means) was rejected with alpha equal to 0.05, the means were compared using the Tukey test (*p* < 0.05).

Finally, the critical levels (CLs) and sufficiency range (SR) of K in the soil and in leaves and pulp were estimated in relation to crop yield, considering the 2 orchards and 2 crop varieties. Before calculating the estimate, the crop yield variable was converted into relative yield (%), considering cultivars, orchards, and crop seasons. The models for estimating the CL and SR values were developed through plateau regression [31,32,33]. For this purpose, the BSQR method was used [32], which quantifies the relationship between the crop yield and the concentration of nutrients in the leaves, pulp, and contents in the soil. Bayesian analysis was used to adjust the parameters of the regression models [45]. In this adjustment, Monte Carlo Simulation with Markov Chains (MCMC) [46] was used with the *Gibbs* sampling algorithm with 20 thousand random designs after a warm-up period of 10 thousand iterations. The modeling was implemented using the “rjags” package from the R statistical environment [44].

The plateau model assumes that crop response increases with the input factor up to a threshold, beyond which additional increases no longer result in significant gains, thereby defining a point of maximum technical efficiency. In turn, the BSQR method extends this approach by modeling different quantiles of the response variable distribution, with emphasis on the upper quantiles (e.g., 0.90), which represent the potential maximum performance of the crop under given conditions. The segmentation allows the identification of changes in the slope of the relationship between the production factor and the response, while the Bayesian framework enables the incorporation of uncertainty and provides more robust parameter estimates, including the change point (breakpoint) [32]. Thus, BSQR not only describes the average trend but also captures the upper boundary of the response, making it particularly useful for defining critical levels (CLs) and sufficiency ranges (SRs) in agricultural systems [31,32,33]. In agronomic terms, the estimated parameters have clear practical interpretations: the breakpoint represents the critical level associated with optimal crop performance, the slope reflects the sensitivity of the response to changes in the factor under limiting conditions, and the plateau indicates the maximum attainable yield or quality under the given conditions. Together, these parameters provide a robust basis for nutrient management recommendations and decision-making in crop production systems.

## 3. Results

### 3.1. Production and Concentrations of K in Soil, Leaves, and Pulp

**Figure 2:**
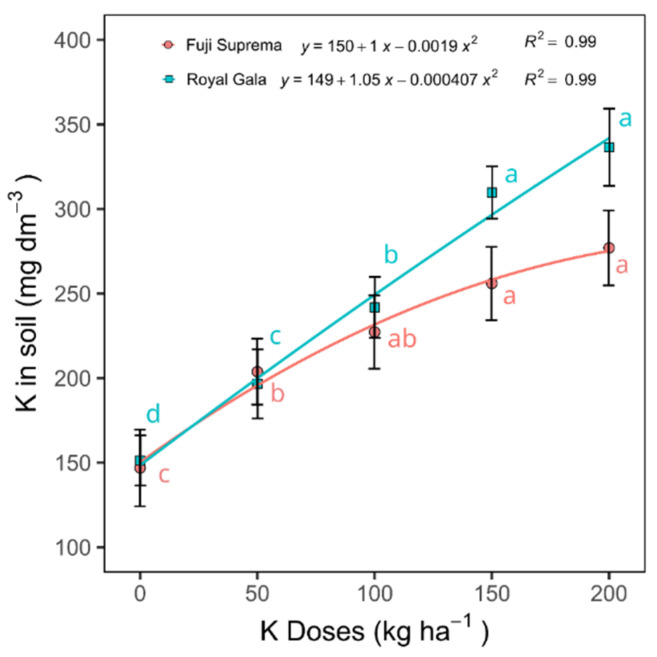
Potassium content in the soil in the 0–0.20 m layer of apple orchards subjected to K doses over four harvests with cultivars “Fuji Suprema” and “Royal Gala”. Lowercase letters compare mean K doses within each cultivar using the Tukey test. Differences were considered significant when the *p*-value < 0.05.

A linear increase in K content in the soil was observed in the 0–0.20 m layer (Figure 2) and 0.20–0.40 m layer (Table S1) with the application of annual K doses.

**Figure 3:**
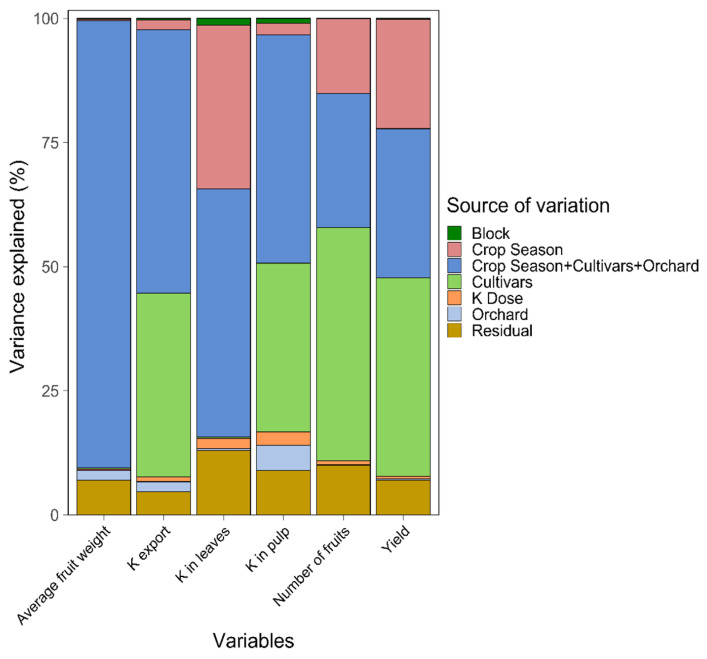
Proportion of variance explained by each source of variation for each response variable. The colors represent the source of variation (K doses, crop season, cultivar, orchard, and interaction: crop season + cultivar + orchard). The response variables are presented on the X axis.

The “cultivar” factor was the variable with the greatest contribution in explaining the variation in yield, K concentration in pulp, and K exported by apples (Figure 3), 40, 34, and 37%, respectively. The second factor with the greatest contribution to apple yield and K concentrations in leaves was the “crop” factor, 22 and 34%, respectively. On the other hand, the interaction of factors “crop season + cultivar + orchard” presented a greater contribution in explaining the variation in mean fruit weight, exported K, and K concentrations in leaves and pulp, 90, 53, and 50%, respectively.

**Figure 4:**
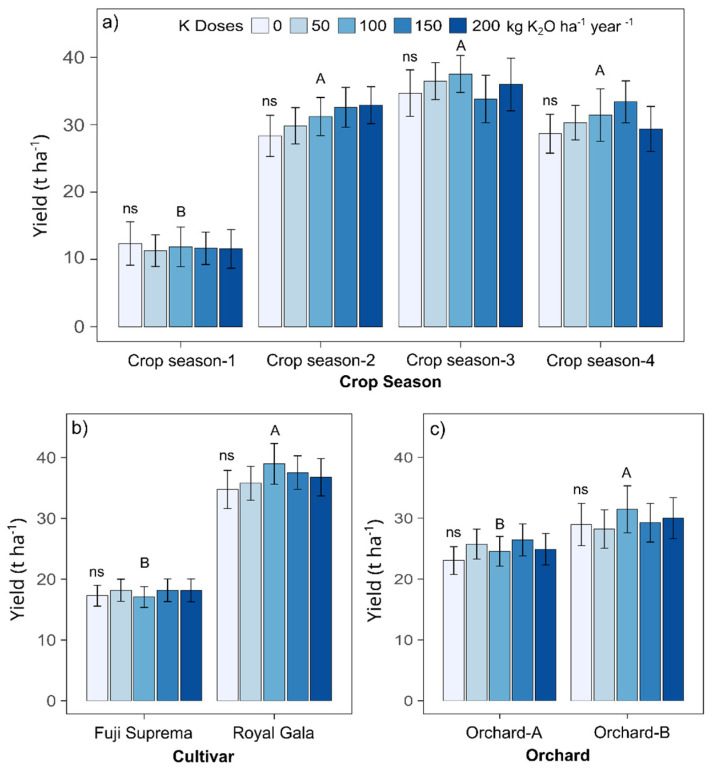
Yield of apples subjected to different doses of K and its relationship with factors “crop season” (**a**), “cultivar” (**b**), and “orchard” (**c**). Uppercase letters compare the effect of K application doses for each factor (crop season, cultivar, and orchard) using Tukey’s test. Differences were considered significant when the *p*-value < 0.05. ns = non-significant at *p* ≥ 0.05.

**Figure 5:**
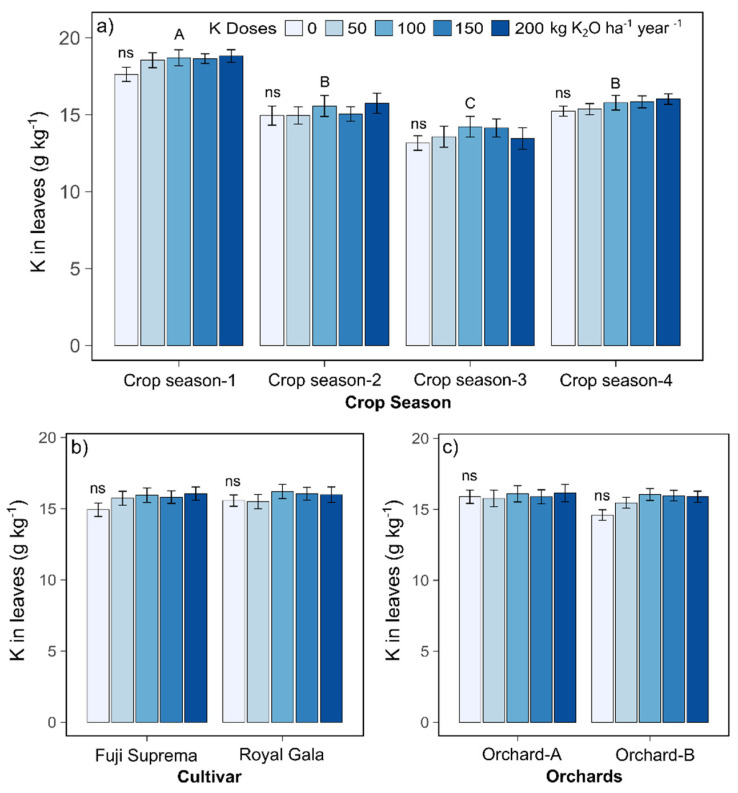
Potassium concentrations in leaves of plants subjected to K doses and their relationship with factors “crop season” (**a**), “cultivar” (**b**), and “orchard” (**c**). Uppercase letters compare the effect of K application doses for each factor (crop season, cultivar, and orchard) using Tukey’s test. Differences were considered significant when the *p*-value < 0.05. ns = non-significant.

The trunk diameter was not significantly affected by K doses, cultivars, or crop seasons (Table S2). Yield did not significantly respond to the K dose when data were pooled across seasons and orchards (Figure 4). However, in crop season 1, cultivar “Fuji Suprema” (11.7 t ha^−1^) and Orchard A (24.9 t ha^−1^) showed the lowest yields compared to the other crop seasons, cultivars, and orchards (Figure 4a–c, respectively). Although an increase in soil K content in “Fuji Suprema” and “Royal Gala”, respectively, was observed with the application of 200 kg K_2_O ha^−1^ year^−1^ (Figure 1), this did not affect K concentrations in the leaves in the four crop seasons (Figure 5a), cultivars (Figure 5b), and orchards (Figure 5c), when the factors were analyzed separately. However, there was a significant difference in K concentrations between crop seasons, regardless of the K doses. The highest concentrations of K in leaves were observed in crop season 1 (18.5 g kg^−1^), followed by crop season 2 (15.7 g kg^−1^), crop season 4 (15.3 g kg^−1^), and crop season 3 (13.7 g kg^−1^) (Figure 5a).

**Figure 6:**
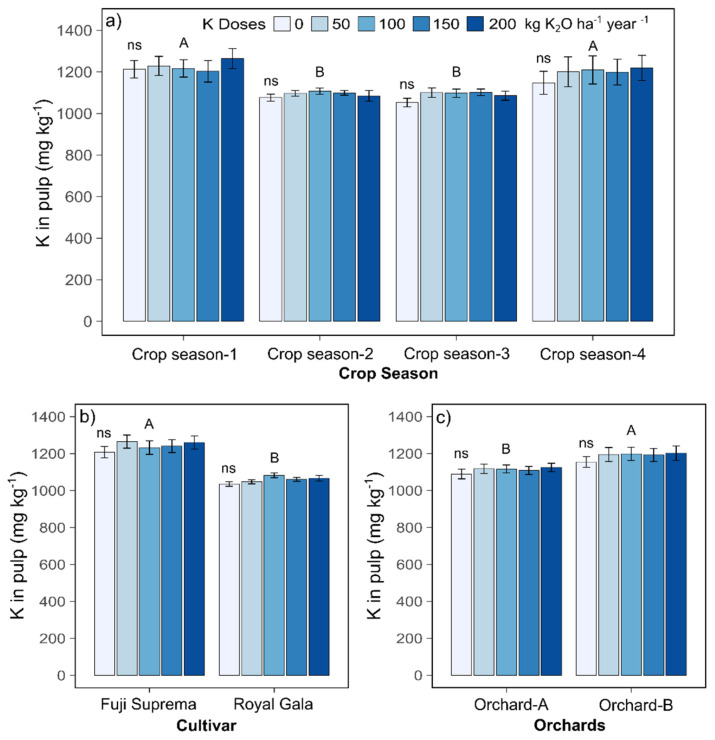
Potassium concentrations in the pulp of apple trees subjected to K doses and their relationship with factors “crop season” (**a**), “cultivar” (**b**), and “orchard” (**c**). Uppercase letters compare the effect of K application doses for each factor (crop season, cultivar, and orchard) using Tukey’s test. Differences were considered significant when the *p*-value < 0.05. ns = non-significant.

A significant effect of K doses on K concentrations in the pulp was observed depending on crop seasons (Figure 6a), cultivars (Figure 6b), and orchards (Figure 6c), when the factors were analyzed separately. The highest concentrations of K in the pulp were observed in crop season 1 and crop season 4, cultivars “Fugi Suprema” and Orchard B, regardless of the K dose (Figure 6a–c, respectively).

### 3.2. Critical Levels and K Sufficiency Ranges

**Figure 7:**
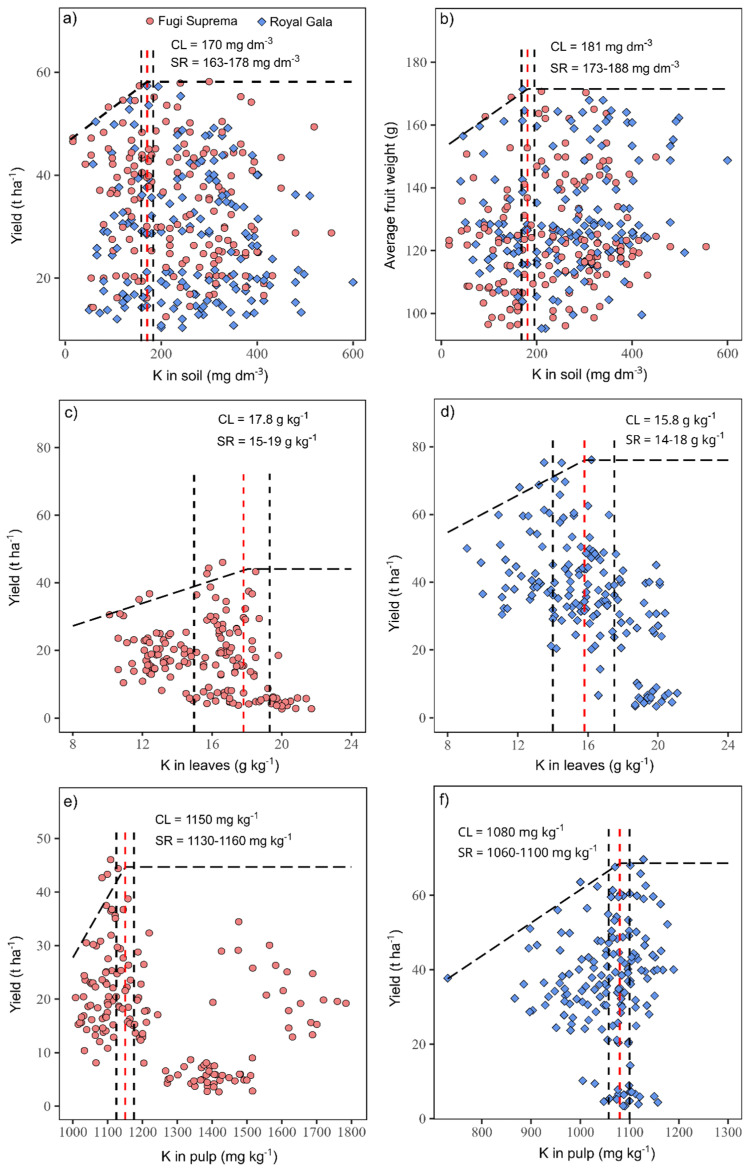
Average critical levels (CLs) and sufficiency range (SR) of K in the soil for the cultivars “*Fuji Suprema*” and “*Royal Gala*” in relation to productivity (**a**) and mean fruit weight of apples (**b**), CL and SR of K in leaves (**c**,**d**) and apple pulp (**e**,**f**) in relation to productivity for both cultivars. The red dashed line represents the CL, and the black dashed line the SR.

The critical concentration (CL) and threshold concentrations (SR) of K in the soil for the apple yield variable were the same for both cultivars (170 mg dm^−3^ and 163–178 mg dm^−3^, Figure 7a). The CL and SR of K in the soil for mean fruit weight were significantly higher than those for yield (181 mg dm^−3^ and 173–188 mg dm^−3^, Figure 7b).

The CL and SR of K in leaves for apple yield presented different values between cultivars; higher for “*Fuji Suprema*” (CL = 17.8 g kg^−1^ and SR = 15–19 g kg^−1^, Figure 7c) than for “*Royal Gala*” (CL = 15.8 g kg^−1^ and SR = 14–18 g kg^−1^, Figure 7d). The CL and SR of K in apple pulp for yield were higher for “*Fuji Suprema*” (CL = 1150 mg kg^−1^ and SR = 1130–1160 mg kg^−1^, Figure 7e) than for “*Royal Gala”* (CL = 1080 mg kg^−1^ and SR = 1060–1100 mg kg^−1^, Figure 7f).

## 4. Discussion

The greatest impact of the cultivar variable on yield, with the highest value (36.5 Mg ha^−1^) observed in Royal Gala, (Figure 4b), probably happened because Fuji is highly susceptible to production alternation (coefficient of variation over the four-harvest period was 48%, Figure S1) [47]. The undesirable effect of alternating production can be reduced by thinning fruits in years with high fruiting; however, this is not enough to avoid the negative effect on the yield accumulated over the years.

Yield influenced by cultivars also affected the concentration of K in leaves and fruits, the amount of K exported, and CL and SR values. A higher yield could be associated with the expected greater redistribution of K from leaves to fruits, reducing levels in leaves [6,48]. Greater productivity also implies a reduction in K levels in the fruit, a consequence of the dilution effect due to the greater fruit load [6]. This occurred in crop seasons 2 and 3, with high fruit yield (Figure 4a) and lower K concentrations in leaves (Figure 5a) and fruits (Figure 5a). However, leaves can exhibit higher concentrations of K when apple crop load is low, resulting from reduced K redistribution, as demonstrated in previous studies [6,49,50], or even when leaf area is limited, which increases nutrient concentration in the tissue [51]. This result is observed in crop season 1, with the lowest fruit yield (Figure 4a) and high levels of K in leaves (Figure 5a).

Crop season was the second variable that most impacted yield and K concentrations in leaves and pulp (Figure 3). This may have happened because of lower volumes or frequency of precipitation, as observed in the months of April, May, July, August, and September in crop season 1 (Figure 1a). This can reduce soil moisture and, consequently, the approach of K to the external surface of the roots [48,52]. In most plants, K approaches the roots by diffusion, which occurs due to the thermal and random movement of the ion in a liquid medium [30]. Therefore, a lower probability of absorption, transport, and allocation of K is expected in organs such as leaves, but also in fruit [6,49].

The crop season was the second factor with the greatest contribution to apple yield (Figure 3), which explains the difference in yield between crop seasons. In the first crop season, low productivity is explained by climatic variables unfavorable to obtaining high apple productivity, such as rainfall and cold times below average, mainly in the months of April, May, June, August, and September (Figure 1a). This behavior is also observed in the fourth crop season (Figure 1d), where a lower volume of rainfall is also observed, resulting in a reduction in productivity in relation to the second and third crop seasons. In this scenario, the effect of the crop season stands out in relation to the effect of the dose of K applied to the orchards.

The increase to 713 plants ha^−1^ (3570 plants ha^−1^ in Orchard B and 2857 plants ha^−1^ in Orchard A) can explain the higher fruit yield of “Fuji” and “Gala” in Orchard B (Figure 4c). In orchards with high plant density, there is greater interception of solar radiation by the plants [53], which can be converted into greater growth and fruit production. Although the K levels in the leaves did not differ between the orchards (Figure 6c), higher K levels in the pulp were observed in Orchard B (Figure 6c). Greater soil exploration by the roots of plants at higher planting densities may increase the absorption of K from the soil [11,23], resulting in greater translocation of K from the soil to the fruits [20,54].

Regarding the fruit yield components, the application of increasing doses of K (up to 200 kg ha^−1^) did not induce a significant increase in the yield and K concentration in apple leaves of “Royal Gala” and “Fuji Suprema” apples across the evaluated crop seasons. This lack of response is highly consistent with the high initial levels of K observed in the 0.00–0.20 m soil layer, which ranged from 143 to 209 mg dm^−3^ (Table 1), values already considered well above the sufficiency threshold for regional standards. K contents in the soils of Orchard A and Orchard B in the 0–20 cm layer were interpreted as “high” (121 to 240 mg dm^−3^ for soils with CTC_pH7.0_ between 15.1 and 30.0 cmol_c_ dm^−3^) by the regional soil chemistry and fertility commission [1]. In these conditions, the soils may have provided the necessary amounts of available K to meet the demand of apple trees [4,48].

The lack of apple yield response to K fertilization under high soil test values observed in our study is consistent with long-term experiments conducted in other temperate regions. For instance, ref. [55] reported that varying soil K levels through fertilization did not induce significant changes in the yield of “Golden Delicious” apple trees in Europe. Similarly, under regional conditions in Southern Brazil, ref. [1] observed that annual K applications over eight seasons failed to increase “Fuji” apple yields, affecting primarily fruit size and tissue nutrient concentrations rather than total production. These findings reinforce the assumption that when orchard soils operate at or above a critical fertility threshold, further K inputs lead to luxury consumption rather than agronomic benefits. Under such conditions, the plant’s root system may have reached a physiological plateau for nutrient uptake, where additional external inputs of K alter soil chemical attributes without translating into biological or agronomic gains. Therefore, these results suggest that in subtropical apple orchards established on soils with naturally high or legacy K fertility, the immediate response to fertilization tends to be negligible, indicating that nutritional maintenance rather than corrective fertilization should be prioritized.

The CL and SF estimated through the BSQR model established a threshold of 170 mg dm^−3^ for K in the soil (Figure 7a). Although the mathematical model provides a precise breakpoint for diagnostic purposes, the high dispersion of data points observed in the relationship between fruit yield and soil K concentrations highlights a significant degree of biological variation under field conditions. This dispersion could be associated with other limiting or co-regulating factors, such as seasonal rainfall distribution, rootstock-scion interactions, and tree age, which often temper the direct effects of soil nutrient availability. Furthermore, the trunk diameter was not significantly affected by K doses, cultivars, or crop seasons, indicating a high structural uniformity among the experimental units and suggesting that the trees’ vegetative vigor remained in a steady state of production, where K availability in the soil was already sufficient to meet the demand of the established plant structure. Consequently, the 170 mg dm^−3^ threshold should not be interpreted as a rigid, but rather as an estimated baseline applicable specifically to orchards managed under similar initial high-K statuses and pedoclimatic conditions.

The calculated sufficiency ranges (SRs) for leaf and fruit pulp K concentrations reflected stable nutritional conditions despite the wide range of applied fertilizer doses. The structural stability of these plant tissue attributes, even under high soil K saturation, may suggest the existence of internal homeostatic regulation mechanisms within the apple trees, limiting excessive luxury consumption to protect fruit quality and shelf-life. Although specific physiological mechanisms—such as soil non-exchangeable K depletion and root uptake dynamics [56]—were not directly monitored in this experiment, the observed behavior is consistent with previous studies in temperate pomology [1,55]. This indicates that under high-K scenarios, tissue analysis serves as a conservative indicator of nutritional status, requiring periodic and systematic monitoring before any drastic management adjustments are made.

Finally, we emphasize that tailoring nutrient recommendations to specific crops, cultivars, and regional conditions could contribute to achieving sustainable yields, optimizing fruit quality, and promoting the rational use of fertilizers. However, developing these frameworks is an ongoing process that depends on the continuous accumulation and compilation of a regional database. While advanced statistical approaches, such as BSQR modeling, offer a valuable tool for identifying critical thresholds (CL and SR), their predictive capacity remains tightly linked to the inherent biological variability of field data. Therefore, well-described datasets integrated with BSQR modeling techniques are the key to providing more reliable and accurate nutrient CL and SR values and, thus, complying with the principles of sustainable pomology.

## 5. Conclusions

This study demonstrates that in high-fertility subtropical soils, annual potassium fertilization increases soil K availability but does not translate into higher apple yields or improved nutritional status in leaves and fruit pulp. Our findings establish that the critical level (CL) of K in the soil is 170 mg dm^−3^ for both “Fuji Suprema” and “Royal Gala” cultivars. Above this threshold, additional K applications are unlikely to provide agronomic benefits, leading to nutrient luxury consumption and potential environmental and economic waste.

Furthermore, this study provides specific diagnostic targets for plant analysis: the CL for leaf K is 17.8 g kg^−1^ for “*Fuji Suprema*” and 15.8 g kg^−1^ for “*Royal Gala*”, reflecting distinct cultivar-specific demands and redistribution patterns. In practical terms, the results of this study indicate that K fertilization strategies in apple orchards may be adjusted according to the K nutritional status of the soil and leaves. Under conditions similar to those evaluated, when K levels exceed the estimated critical thresholds, maintenance fertilization may be reduced or temporarily suspended, provided that such decisions are supported by continuous nutritional monitoring and consideration of orchard-specific conditions. The proposed thresholds should be interpreted as reference values to support decision-making in K management, contributing to more efficient fertilizer use and the maintenance of orchard productivity under subtropical conditions.

## Figures and Tables

**Table 1:** Values of cation exchange capacity (CEC) and exchangeable K in the soil of the two orchards and cultivars in the year the experiment was implemented in 2002.

Cultivar	Orchard	Layer (m)	Soil Properties			
			pH in H_2_O	SOM (%)	CEC (cmol_c_ kg^−1^)	K (mg dm^−3^)
Fuji Suprema	A	0.00–0.20	6.5	4.3	19.71 (±3.10)	160.50 (±21.43)
		0.20–0.40	5.9	3.7	21.50 (±4.85)	110.75 (±23.89)
	B	0.00–0.20	6.5	4.2	17.76 (±2.15)	165.25 (±13.93)
		0.20–0.40	5.7	4.1	19.14 (±2.10)	65.00 (±8.71)
Royal Gala	A	0.00–0.20	6.8	4.5	20.03 (±5.05)	143.25 (±11.35)
		0.20–0.40	6.2	4.1	21.78 (±1.89)	117.25 (±38.05)
	B	0.00–0.20	6.1	4.8	17.26 (±3.25)	209.25 (±62.40)
		0.20–0.40	5.8	4.6	18.56 (±4.10)	117.50 (±38.05)

## Data Availability

The data presented in this study are available on request from the corresponding author. The data are not publicly available due to privacy concerns.

## Supplementary Materials

The following supporting information can be downloaded at: https://www.mdpi.com/article/10.3390/plants15121866/s1. Figure S1: Yield of apples subjected to different doses of K and its relationship with factors “crop season” and “cultivar”. Table S1: Potassium content (mg dm^−3^) in the soil in the 0–0.20 m soil depth in apple orchards (A and B), subjected to K doses over four harvests with cultivars “Fuji Suprema” and “Royal Gala”. Table S2: Trunk diameter of “Fuji Suprema” and “Royal Gala” apple trees cultivated in two orchards (A and B) and submitted to potassium fertilization for four seasons.

## References

[1] Nava G., Dechen A.R. (2009). Long-term annual fertilization with nitrogen and potassium affect yield and mineral composition of ‘fuji’ apple. Sci. Agric..

[2] CQFS-RS/SC—Comissão de Química e Fertilidade Do Solo—RS/SC (2016). Manual de Adubação e Calagem Para os Estados do Rio Grande do Sul e Santa Catarina.

[3] Meurer E.J., Rosso J.I. (1997). Kinetics of potassium release from soils of the state of Rio Grande do Sul, Brazil. Rev. Bras. Ciência Do Solo.

[4] Mota M., Martins M.J., Policarpo G., Sprey L., Pastaneira M., Almeida P., Maurício A., Rosa C., Faria J., Martins M.B. (2022). Nutrient Content with Different fertilizer management and influence on yield and fruit quality in apple cv. Gala. Horticulturae.

[5] Nachtigall G.R., Carraro H.R., Alleoni L.R.F. (2007). Potassium, calcium, and magnesium distribution in an Oxisol under long-term potassium-fertilized apple orchard. Commun. Soil Sci. Plant Anal..

[6] Csihon Á., Gonda I., Sipos M., Holb I.J. (2024). Impacts of N-P-K-Mg Fertilizer Combinations on Tree Parameters and Fungal Disease Incidences in Apple Cultivars with Varying Disease Susceptibility. Plants.

[7] Xu X., Du X., Wang F., Sha J., Chen Q., Tian G., Zhu Z., Ge S., Jiang Y. (2020). Effects of Potassium Levels on Plant Growth, Accumulation and Distribution of Carbon, and Nitrate Metabolism in Apple Dwarf Rootstock Seedlings. Front. Plant Sci..

[8] Solhjoo S., Gharaghani A., Fallahi E. (2017). Calcium and potassium foliar sprays affect fruit skin color, quality attributes, and mineral nutrient concentrations of ‘Red Delicious’ apples. Int. J. Fruit Sci..

[9] Musacchi S., Serra S. (2018). Apple fruit quality: Overview on pre-harvest factors. Sci. Hortic..

[10] van Raij B., Yamada T., Igue K., Muzilii O., Usherwood N.R. (1982). Disponibilidade de potássio em solos do Brasil. Potássio na Agricultura Brasileira Piracicaba.

[11] Kuzin A., Solovchenko A. (2021). Essential Role of Potassium in Apple and Its Implications for Management of Orchard Fertilization. Plants.

[12] Kopsell D.E., Kopsell D.A., Sams C.E., Barickman T.C. (2013). Ratio of calcium to magnesium influences biomass elemental accumulations and pigment concentrations in kale. J. Plant Nutr..

[13] Nguyen H., Maneepong S., Suraninpong P. (2017). Effects of potassium, calcium and magnesium ratios in soil on their uptake and fruit quality of pummelo. J. Agric. Sci..

[14] Freitas S.T., Mitcham E.J., Janick J. (2012). Factors involved in fruit calcium deficiency disorders. Horticultural Reviews.

[15] Corrêa T.R., Steffens C.A., Amarante C.V.T., Miqueloto A., Brackmann A., Ernani P.R. (2017). Multivariate analysis of mineral content associated with flesh browning disorder in ‘Fuji’ apples produced in Southern Brazil. Bragantia.

[16] Miqueloto A., Amarante C.V.T., Steffens C.A., Santos A., Heinzen A.S., Miqueloto T., Strauss R., Finger F.L., Picoli E.A.T., Souza G.A. (2018). Mechanisms regulating fruit calcium content and susceptibility to bitter pit in cultivars of apple. Acta Hortic..

[17] Parent L.-É. (2011). Diagnosis of the nutrient compositional space of fruit crops. Rev. Bras. Frutic..

[18] Prado R.M., Rozane D.E. (2020). Leaf analysis as diagnostic tool for balanced fertilization in tropical fruits. Fruit Crops.

[19] Paula B.V.D., Rozane D.E., Melo G.W.B.D., Natale W., Marques A.C.R., Brunetto G. (2021). Kinetic parameters estimation for increasing the efficiency of nutrient absorption in fruit trees. Rev. Bras. Frutic..

[20] Tagliavini M., Scandellari F. (2012). Methodologies and concepts in the study of nutrient uptake requirements and partitioning in fruit trees. Acta Hortic..

[21] Shahkoomahally S., Chaparro J.X., Beckman T.G., Sarkhosh A. (2020). Influence of rootstocks on leaf mineral content in the subtropical peach cv. UFSun. HortScience.

[22] Zhou Q., Melgar J.C. (2020). Tree Age Influences Nutrient Partitioning among Annually Removed Aboveground Organs of Peach. HortScience.

[23] Jungk A., Claassen N. (1986). Availability of phosphate and potassium as the result of interactions between root and soil in the rhizosphere. J. Pant Nutr. Soil Sci..

[24] Yang Y., Yang Z., Yu S., Chen H. (2019). Organic acids exuded from roots increase the available potassium content in the rhizosphere soil: A rhizobag experiment in Nicotiana tabacum. HortScience.

[25] Volf M.R., Guimarães T.M., Scudeletti D., Cruz I.V., Rosolem C.A. (2018). Potassium dynamics in ruzigrass rhizosphere. Rev. Bras. Ciênc. Solo.

[26] Hinsinger P., Jaillard B. (1993). Root-induced release of interlayer potassium and vermiculitization of phlogopite as related to potassium depletion in the rhizosphere of ryegrass. Eur. J. Soil Sci..

[27] Bortoluzzi E.C., Moterle D.F., Rheinheimer D.S., Casali C.A., Melo G.W., Brunetto G. (2012). Mineralogical changes caused by grape production in a regosol from subtropical Brazilian climate. J. Soils Sediments.

[28] Zhang Q., Zhou B., Li M., Wei Q., Han Z. (2018). Multivariate analysis between meteorological factor and fruit quality. J. Integr. Agric..

[29] Lawrence B.T., Melgar J.C. (2018). Variable fall climate influences nutrient resorption and reserve storage in young peach trees. Front. Plant Sci..

[30] Hahn L., Basso C., Moura-Bueno J.M., Argenta L.C., Toselli M., Carranca C., Rech M., Hahn I.S., Brunetto G. (2023). Yield Prediction Models for ‘Royal Gala’ and ‘Fuji Suprema’ Apple Varieties Cultivated under a Subtropical Climate. Agronomy.

[31] Makowski D., Doré T., Monod H. (2007). A new method to analyse relationships between yield components with boundary lines. Agron. Sustain. Dev..

[32] Theobald C.M., Talbot M. (2002). The Bayesian choice of crop variety and fertilizer dose. J. R. Stat. Soc. Ser. C Appl. Stat..

[33] Liang Z., Qian S.S., Wu S., Chen H., Liu Y., Yu Y., Yi X. (2019). Using Bayesian change point model to enhance understanding of the shifting nutrients-phytoplankton relationship. Ecol. Model..

[34] Andrade C.B., Comin J.J., Moura-Bueno J.M., Brunetto G. (2023). Obtaining reference values for nutrients in vineyard soils through boundary line approach using Bayesian segmented quantile regression on commercial farm data. Eur. J. Agron..

[35] Hindersmann J., Moura-Bueno J.M., Brunetto G., Tiecher T., Natale W., De Lima Neto A.J., Klock L.H., Bernardt E., Silva J.H.S., Nava G. (2026). Proposing Nutrient Reference Values in Peach Leaves Based on Bayesian Segmented Quantile Regression. J. Soil Sci. Plant Nutr..

[36] Stefanello L., Schwalbert R., Schwalbert R., Tassinari A., Garlet L., De Conti L., Ciotta M., Ceretta C., Ciampitti I., Brunetto G. (2023). Phosphorus critical levels in soil and grapevine leaves for South Brazil vineyards: A Bayesian approach. Eur. J. Agron..

[37] Alvares C.A., Stape J.L., Sentelhas P.C., Gonçalves J.L.M., de Moraes Gonçalves J.L., Sparovek G. (2013). Köppen’s climate classification map for Brazil. Meteorol. Z..

[38] Soil Survey Staff (2014). Keys to Soil Taxonomy.

[39] Epagri-Empresa de Pesquisa Agropecuária e Extensão Rural de Santa Catarina (2022). A Cultura da Macieira.

[40] EMBRAPA, Brazilian Agricultural Research Corporation (2009). Manual of Chemical Analysis of Soils, Plants and Fertilizers.

[41] Teixeira P.C., Donagemma G.K., Fontana A., Teixeira W.G. (2017). Manual of Soil Analysis Methods.

[42] EPAGRI—Empresa de Pesquisa Agropecuária e Extensão Rural de Santa Catarina (2020). Santa Catarina Environmental Variables Database.

[43] Ebert A., Petri J.L., Bender R.J., Braga H.J. (1986). First experiences with chill-unit models in Southern Brazil. Acta Hortic..

[44] R Core Team (2021). R: A Language and Environment for Statistical Computing.

[45] Kruschke J.K., Liddell T.M. (2018). Bayesian data analysis for newcomers. Psychon. Bull. Rev..

[46] Gelman A., Hill J. (2007). Data Analysis Using Regression and Multilevel/Hierarchical Models.

[47] Ernani P.R., Dias J., Flore J.A. (2002). Annual additions of potassium to the soil increased apple yield in Brazil. Commun. Soil Sci. Plant Anal..

[48] Nachtigall G.R., Dechen A.R. (2006). Seasonality of nutrients in leaves and fruits of apple trees. Sci. Agric..

[49] Mészáros M., Hnátková H., Conka P., Námstek J. (2021). Linking Mineral Nutrition and Fruit Quality to Growth Intensity and Crop Load in Apple. Agronomy.

[50] Čonka P., Bělíková H., Mészáros M., Kurešová G., Náměstek J. (2017). Evaluation of seasonal variation in mineral composition of leaves and fruits of Malus domestica Borkh. ‘Golden Delicious’ throughout growing season. Vědecké Práce Ovocnářské.

[51] Aichner M., Stimpfl E. (2002). Seasonal pattern and interpretation of mineral nutrient concentrations in apple leaves. Acta Hortic..

[52] Rosolem C.A., Mateus G.P., Godoy L.J.G., Feltran J.C., Brancalião S.R. (2003). Root morphology and potassium supply to pearl millet roots as affected by soil water and potassium contents. Rev. Bras. Cienc. Solo.

[53] Bhat K.K.S. (1983). Nutrient inflows into apple roots. Plant Soil.

[54] Hinsinger P., Plassard C., Tang C., Jaillard B. (2003). Origins of root-mediated pH changes in the rhizosphere and their responses to environmental constraints: A review. Plant Soil.

[55] Szewczuk A., Komosa A., Gudarowska E. (2008). Effect of soil potassium levels and different potassium fertilizer forms on yield and storability of ‘golden delicious’ apples. Acta Sci. Pol. Hortorum Cultus.

[56] Steiner F., Lana M.D.C. (2018). Contribution of non-exchangeable K in soils from Southern Brazil under potassium fertilization and successive cropping. Rev. Ciênc. Agron..

