# Supplementary material for: Yield Response and Calibration of Critical Potassium Levels in Soil, Leaves, and Fruit Pulp of “Royal Gala” and “Fuji Suprema” Apples
Source: Plants (Basel). 2026 Jun 16;15(12):1866. doi: 10.3390/plants15121866 (PMC13306360; doi:10.3390/plants15121866)
Supplement: Supplementary file 1 [file plants-15-01866-s001.zip › plants-4229648-supplementary.pdf]

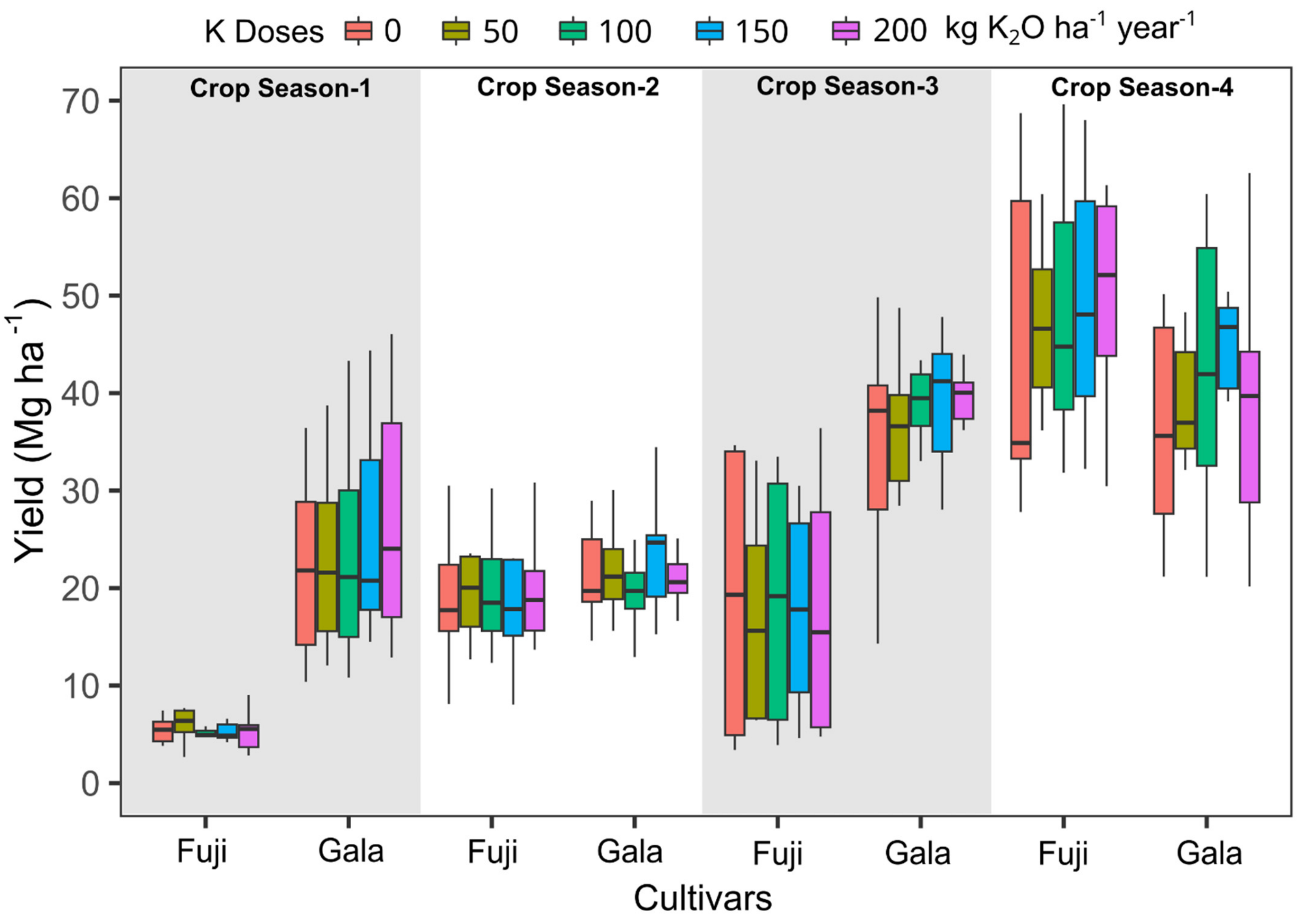

Figure S1: Yield of apples subjected to different doses of K and its relationship with factors “crop season” and “cultivar”.

Table S1. Potassium content (mg dm^{-3}) in the soil in the 0-0.20 m soil depth in apple orchards (A and B), subjected to K doses over four harvests with cultivars 'Fuji Suprema' and 'Royal Gala'.

Cultivar	Orchard	Layer (m)	K doses (kg ha^{-1})	Crop seasons 2004	Crop seasons 2006
Fuji Suprema	A	0.00 – 0.20	0	202	175
			50	275	232
			100	291	355
			150	319	380
			200	374	429
		0.20 – 0.40	0	193	133
			50	234	232
			100	188	306
			150	232	350
			200	233	352
	B	0.00 – 0.20	0	110	98
			50	173	204
			100	293	321
			150	319	209
			200	325	407
		0.20 – 0.40	0	84	58
			50	135	100
			100	137	131
			150	154	308
			200	177	231
Royal Gala	A	0.00 – 0.20	0	182	164
			50	203	242
			100	276	308
			150	333	356
			200	396	429
		0.20 – 0.40	0	134	132
			50	195	180
			100	219	301
			150	298	301
			200	329	296
	B	0.00 – 0.20	0	154	195
			50	268	312
			100	306	360
			150	355	424
			200	387	470
		0.20 – 0.40	0	137	101
			50	122	186
			100	167	290
			150	253	363
			200	235	339

Table S2. Trunk diameter of 'Fuji Suprema' and 'Royal Gala' apple trees cultivated in two orchards (A and B) and submitted to potassium fertilization for four seasons.

Orchard	Cultivar	Crop season	Trunk diameter ± sd (cm)
A	Fuji Suprema	1	13,6±0,7
		2	15,3±0,9
		3	18,1±1,0
		4	19,7±1,7
		Average	16,7±1,1
	Royal. Gala	1	13,9±0,6
		2	15,8±0,9
		3	19,0±1,0
		4	20,0±1,2
		Average	17,2±0,9
B	Fuji Suprema	1	12,4±0,4
		2	13,3±0,5
		3	15,2±0,7
		4	15,9±0,9
		Average	14,2±0,6
	Royal. Gala	1	15,2±0,9
		2	15,9±1,0
		3	18,0±1,2
		4	18,7±1,2
		Average	16,9±1,1
